# The N-Terminal Membrane-Spanning Domain of the *Escherichia coli* DNA Translocase FtsK Hexamerizes at Midcell

**DOI:** 10.1128/mBio.00800-13

**Published:** 2013-12-03

**Authors:** Paola Bisicchia, Bradley Steel, Mekdes H. Mariam Debela, Jan Löwe, David Sherratt

**Affiliations:** Department of Biochemistry^a^; Department of Physics,^b^ University of Oxford, Oxford, United Kingdom; MRC Laboratory of Molecular Biology, Cambridge Biomedical Campus, Cambridge, United Kingdom^c^

## Abstract

Bacterial FtsK plays a key role in coordinating cell division with the late stages of chromosome segregation. The N-terminal membrane-spanning domain of FtsK is required for cell division, whereas the C-terminal domain is a fast double-stranded DNA (dsDNA) translocase that brings the replication termination region of the chromosome to midcell, where it facilitates chromosome unlinking by activating XerCD-*dif* site-specific recombination. Therefore, FtsK coordinates the late stages of chromosome segregation with cell division. Although the translocase is known to act as a hexamer on DNA, it is unknown when and how hexamers form, as is the number of FtsK molecules in the cell and within the divisome. Using single-molecule live-cell imaging, we show that newborn *Escherichia coli* cells growing in minimal medium contain ~40 membrane-bound FtsK molecules that are largely monomeric; the numbers increase proportionately with cell growth. After recruitment to the midcell, FtsK is present only as hexamers. Hexamers are observed in all cells and form before any visible sign of cell constriction. An average of 7 FtsK hexamers per cell are present at midcell, with the N-terminal domain being able to hexamerize independently of the translocase. Detergent-solubilized and purified FtsK N-terminal domains readily form hexamers, as determined by *in vitro* biochemistry, thereby supporting the *in vivo* data. The hexameric state of the FtsK N-terminal domain at the division site may facilitate assembly of a functional C-terminal DNA translocase on chromosomal DNA.

## IMPORTANCE

In the rod-shaped bacterium *Escherichia coli*, more than a dozen proteins act at the cell center to mediate cell division, which initiates while chromosome replication and segregation are under way. The protein FtsK coordinates cell division with the late stages of chromosome segregation. The N-terminal part of FtsK is membrane embedded and acts in division, while the C-terminal part forms a hexameric ring on chromosomal DNA, which the DNA can translocate rapidly to finalize chromosome segregation. Using quantitative live-cell imaging, which measures the position and number of FtsK molecules, we show that in all cells, FtsK hexamers form only at the cell center at the initiation of cell division. Furthermore, the FtsK N-terminal portion forms hexamers independently of the C-terminal translocase.

## INTRODUCTION

The foundations of our understanding of bacterial cell division were laid down with the early isolation of temperature-sensitive mutants in cell division (1, 2). Subsequent genetic analyses identified more than a dozen *Escherichia coli* cell division genes with a clear pattern of genetic dependencies (reviewed in references 3 and 4). For example, recruitment of the FtsZ tubulin is required for the recruitment of most of the other divisome proteins. Similar work with other bacteria has identified a core of essential conserved division proteins (5). Biochemical, structural, and cell biological analysis then provided many details of cell division, although biochemical mechanistic details remain largely lacking (6, 7).

**FIG 1:**
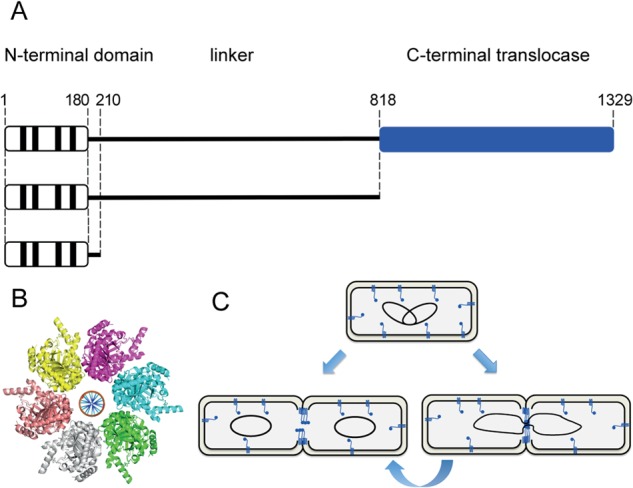
Schematic representation of FtsK and its localization during cell division. (A) Diagrams representing the *E. coli* FtsK protein. The top diagram depicts the wild-type protein, while the bottom diagrams represent the different deletion mutants analyzed in this work. Numbers indicate the positions of amino acid residues. Vertical black bars represent transmembrane segments contained in the N-terminal domain. The C-terminal translocase domain is represented in blue. (B) Crystal structure of hexameric *Pseudomonas aeruginosa* FtsK_C_Δγ containing modeled DNA (31). (C) Schematic of FtsK localization throughout the cell cycle in the absence and presence of chromosome dimers (left and right diagrams, respectively). In a growing cell (top row), inner-membrane-associated FtsK is distributed throughout the cell. In dividing cells (second row), FtsK is recruited to divisomes in all cells, independently of the presence of chromosome dimers. Until this work, the number of cellular FtsK molecules and their oligomeric state as a function of cell cycle have not been known.

The work reported here focuses on FtsK, a large 1,329-amino-acid integral membrane protein that is recruited to the developing divisome soon after FtsZ but acts late in cell division, during which it coordinates cell division with the late stages of chromosome segregation (8–12) (Fig. 1). An ~200-amino-acid N-terminal domain (FtsK_N_), which has four or five membrane-spanning segments, appears to function in cytokinesis by interacting with other division proteins and stabilizing the divisome complex (13–16). In *E. coli*, this domain is connected to an ~550-amino-acid DNA translocase domain (FtsK_C_) by an ~650-amino-acid proline- and glycine-rich linker, which is required for normal FtsK activity (10, 17, 18), with linker size varying in different bacteria.

It has generally been believed that the C-terminal translocase functions only in the minority of cells that contain chromosome dimers or catenanes or which are unable to complete normal chromosome segregation because of DNA damage and/or a failure to complete replication (10, 19–25).

However, this view has been challenged by recent work that indicates that *E. coli* FtsK translocation functions in all cells to determine the normal segregation pattern of the last loci to be segregated prior to completion of cell division (26). A related FtsK ortholog, SpoIIIE, is responsible for translocating much of the *Bacillus subtilis* chromosome into developing spores (27–30).

Hexameric FtsK translocase acts directionally on the chromosome to ensure that its translocation brings the terminus region *dif* site to the septum, where *dif*-bound XerCD recombinase is activated to mediate dimer resolution and decatenation (31, 32). Previous work using the strain and conditions used here has shown that FtsK is recruited to midcell soon after FtsZ and well before constriction at midcell is initiated (33). Nevertheless, it has been proposed that a functional hexameric translocase forms only at a constricting septum, where the high local concentrations of protein and DNA favor the maintenance of the hexameric state by ensuring a high “on” rate of translocase monomers to form hexamers on DNA (34). In order to address the oligomeric state of FtsK after it is recruited to midcell as part of the developing divisome and when it is present elsewhere in the cell, we used quantitative live-cell imaging. We showed that all detectable FtsK molecules present at midcell in dividing cells were hexameric, whereas those present before divisome formation appeared to be largely monomeric and distributed throughout the inner cell membrane. The N-terminal membrane-spanning domain was sufficient for hexamer formation *in vivo*, and purified protein confirmed the hexameric state. We showed 1 to 7 FtsK hexamers present at midcell in dividing cells, dependent on the stage of divisome assembly, representing 10 to 45% of the cellular FtsK pool. Two other divisome proteins, ZapC and TolQ, which do not interact with FtsK (17, 35), were not present at midcell as discrete oligomers, whereas FtsQ, which interacts with FtsK and whose positioning at the cell center is FtsK dependent (17, 35), was recruited to form hexameric clusters at midcell.

## RESULTS AND DISCUSSION

### FtsK is present as hexamers at midcell.

In order to determine the stoichiometry and architecture of the FtsK translocase in live *E. coli* cells, we constructed C-terminal fusions of the fluorescent protein YPet to wild-type FtsK and truncated derivatives of it expressed from the endogenous wild-type chromosomal locus. YPet has been used in previous experiments to determine protein stoichiometries and architecture because of its brightness and fast maturation (36). Cells were grown in minimal glycerol medium, under conditions in which cell cycle parameters are well defined (33), and the fusions were fully functional in all available assays (see Text S1 [materials and methods] in the supplemental material). Septation, assessed by the initiation of constriction, initiates ~77 min after birth in these cells, which divide every 100 min, with FtsK appearing soon after FtsZ, which first appears as a ring at midcell at ~48 min (33).

**FIG 2:**
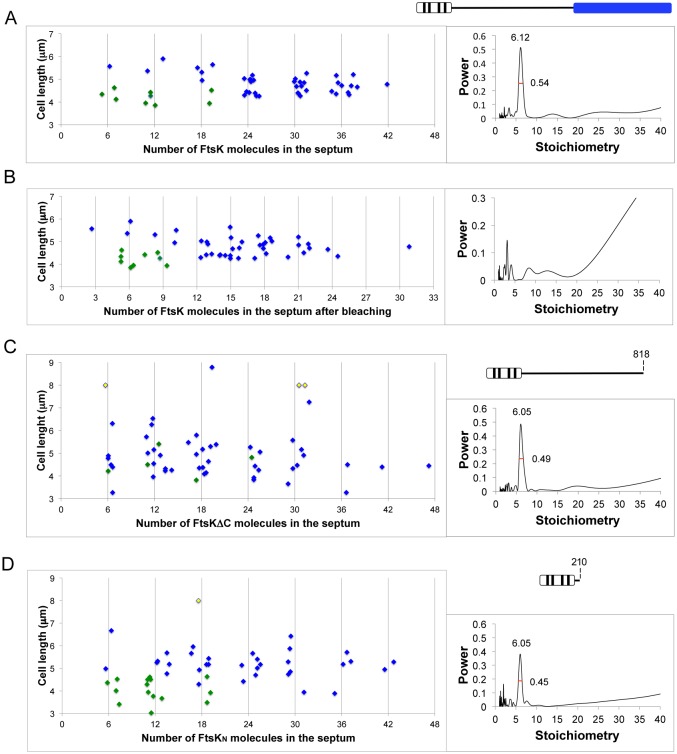
Stoichiometry of FtsK at midcell. (A, C, and D) Stoichiometry of YPet derivatives of midcell FtsK (A), FtsKΔ_C_ (C), and FtsK_N_ (D). (B) Fluorophore stoichiometry after ~50% of the fluorophores had been bleached. Green symbols represent unconstricted cells, blue symbols represent constricted cells, and yellow symbols represent filaments whose length exceeds 9 µm. Plots on the right show the periodicity derived from power spectra of the data displayed in the adjacent graphs, with the peak width at half-maximal height also shown. Schematics of wild-type and mutated variants of FtsK are shown above the graphs.

Cells exhibiting fluorescent FtsK at midcell were imaged by widefield microscopy in an OMX V3 Blaze microscope, and the data were analyzed by a custom-written Matlab code that fitted an elliptical Gaussian curve over the strong fluorescent signal in the region of the prospective division site (see Text S1 in the supplemental material). After background was subtracted, the integrated fluorescence within the fitted Gaussian curve was divided by the intensity of a single YPet fluorophore, determined from analyzing cells expressing 1 to 10 LacY-YPet monomeric molecules, thereby yielding the number of FtsK-YPet fluorophores at midcell (Fig. 2A, left). We found 6 to 42 wild-type FtsK molecules at midcell (mean, 25), dependent on cell length and degree of septal constriction. A power spectrum of the measured stoichiometries of FtsK at midcell was used to highlight periodicities in the measured brightness values. The spectrum showed a single dominant peak of stoichiometry of 6.12, with a peak half width (half maximum) of 0.54, indicating that FtsK is present at midcell as hexamers and/or multiples of hexamers.

FtsK hexamers were present at midcell in all cells approaching septation and not just in the subpopulation (~15%) of cells in which chromosome dimers were expected to form (19, 23, 24). This results shows that FtsK is able to hexamerize independently of chromosome dimer formation and is consistent with the results of experiments that show that FtsK accesses chromosomal DNA in all cells rather than in the minority subpopulation that contains chromosome dimers (26). One to three hexamers were present before visible constriction (Fig. 2A, green, and see Fig. 4C). Four to seven hexamers were present relatively early in constriction (Fig. 2A, blue), but this number was reduced to one hexamer toward the end of constriction (see Fig. 4C). Since hexameric stoichiometry was retained until the final stages of division, molecules were released as hexamers; alternatively, if hexamers disassemble, then monomers and other small oligomers must diffuse away rapidly.

In order to confirm the validity of the methodology and to ensure that the periodicity of 6 was not an artifact of the analytical method, we reanalyzed cells in which the total fluorescent signal had been bleached to ~50% of the initial value (Fig. S1B). All signs of hexameric periodicity were lost, and we observed the expected binomial distribution of fluorophore numbers centered at multiples of 3 (Fig. 2B). This validates the methodology and the results obtained for FtsK and shows that this is a robust method for determining the stoichiometry of other divisome proteins.

### The C-terminal FtsK translocase is not required for hexamerization.

The ability of an isolated FtsK C-terminal translocase domain (FtsK_C_) to form functional hexamers has been established from *in vitro* analysis (31, 37). Furthermore, it has been proposed that hexamerization is promoted at a constricting division septum because it is only then that FtsK hexamerizes and loads onto chromosomal DNA because of the high local concentrations of FtsK and DNA there (34). To test whether the translocase is responsible for promoting and maintaining the hexameric state of FtsK *in vivo*, we determined the stoichiometry of an FtsK derivative lacking the C-terminal domain (FtsKΔ_C_; amino acids 1 to 818) and one containing only the N-terminal domain (FtsK_N_; amino acids 1 to 210). FtsKΔ_C_-YPet cells showed the same periodicity of stoichiometries at midcell as its wild-type counterpart (stoichiometry of 6.05; half width [half maximum] of the peak, 0.49) (Fig. 2C). Cells mutated for the translocase show higher filamentation and reduced viability, as reported previously (9, 13, 14) (Fig. 2C, left).

Next, we analyzed FtsK_N_-YPet, which also showed a periodicity of 6 despite increased length and filamentation and reduced viability; these phenotypes are consistent with previous results (9, 13, 14, 38) (Fig. 2D, left). We conclude therefore that FtsK_N_ and the C-terminal translocase hexamerize independently.

The finding that FtsK hexamers are always present at prospective division sites as a consequence of hexamerization of the N-terminal domain indicates that this will provide a chelate effect that should promote hexamerization of the C-terminal translocase immediately after FtsK is recruited to midcell and before constriction is initiated. This finding contrasts with the proposal that the FtsK translocase may form only hexamers after cell constriction is initiated, thereby forming a small-volume compartment that has a high local concentration of DNA and protein (34). Our finding is consistent with the demonstration that an active hexameric FtsK translocase can access chromosomal DNA in all cells (26), because at the time of hexamer formation, chromosome segregation will not have been completed in most cells (33).

We do not know if the presence of FtsK_N_ and FtsK hexamers at midcell arises simply because of the high local concentration there or whether there are other processes that direct hexamerization. If we assume that no other process is responsible for keeping FtsK in hexameric form at midcell, the dissociation rate of FtsK_N_ hexamers into monomers is expected to be the same for the molecules present at midcell and elsewhere in the cell, whereas that of at least the translocating molecules of FtsK may be lower at midcell because of the chelate effect of the translocase hexamerizing on DNA. We note that when a single hexamer is present at midcell, the local concentration of monomers is not expected to be significantly higher than that of the noncentral pool, suggestive of additional processes favoring the maintenance of hexamers at midcell.

### Purified FtsK_N_ forms hexamers *in vitro.*

**FIG 3:**
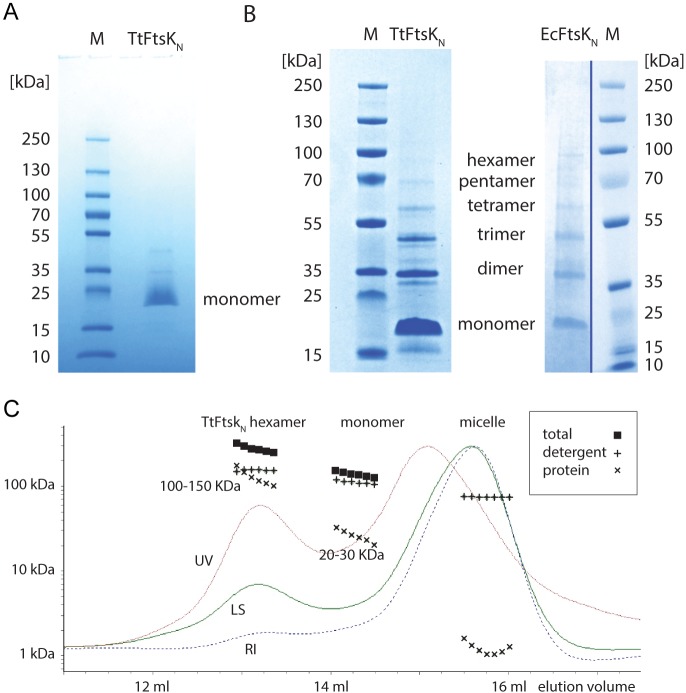
The N-terminal domains of FtsK from *Escherichia coli* (EcFtsK_N_) and *Thermoanaerobacter tengcongensis* (TtFtsK_N_) form hexamers *in vitro*. (A) Four to 12% bis-Tris Coomassie blue-stained SDS-PAGE gel of TtFtsK_N_ after the first Talon cobalt affinity chromatography purification from membrane extract in a high detergent concentration. A single band at ~20 kDa is visible, in agreement with the expected size of the protein. (B) Sample fractions from the Superdex S-200 size exclusion second-purification steps, with a lower detergent concentration, analyzed on 12% Coomassie blue-stained SDS-PAGE gels. Several bands larger than the monomer appear, with the hexamer visible and indicating stable oligomerization of both EcFtsK_N_ and TtFtsK_N_. Note that the proteins cannot be entirely unfolded since otherwise they would not form oligomers, and hence the oligomers run slightly faster than expected for an SDS-unfolded sample. (C) Size exclusion chromatography-multiangle light scattering (SEC-MALS) analysis of TtFtsK_N_. Using a Superdex S-200 size exclusion column, samples were detected using UV absorption at 280 nm (UV), light scattering (LS), and a refractive index (RI). The UV profile shows a higher-molecular-weight peak and a lower-molecular-weight peak. Using MALS and DDM detergent micelle samples, the molecular weights of the two peaks minus detergent contribution could be determined to be the hexamer and monomer, with average masses of 100 to 150 kDa and 20 to 30 kDa, respectively. Figure S2B shows the detergent micelle traces, which were omitted here for clarity. Lanes M, molecular mass markers.

The N-terminal domains of FtsK from *E. coli* (EcFtsK_N_) and *Thermoanaerobacter tengcongensis* (TtFtsK_N_) were purified to homogeneity using DDM (*n*-dodecyl β-d-maltopyranoside) detergent solubilization, a C-terminal 10-histidine tag, metal affinity chromatography, and size exclusion chromatography (SEC) (Fig. 3A; Text S1). TtFtsK_N_ shares only 30% amino acid similarity with the EcFtsK_N_ protein (Fig. S2A), and both isolated domains were well behaved during purification and characterization. When detergent concentration was lowered during the purification, distinct oligomerization bands appeared on SDS-PAGE gels for both proteins (Fig. 3B). In both instances, these clearly correspond to dimers up to hexamers. The oligomers run faster through the gel, as would be expected for similarly sized, fully unfolded proteins, giving rise to apparent molecular weights that are lower than expected. This behavior is normal, as the protein clearly cannot be fully unfolded, since otherwise oligomerization would not be observed.

We then employed SEC combined with multiple-angle light scattering (SEC-MALS) in order to obtain reliable mass estimates of the monomeric and oligomerized protein species for TtFtsK_N_, without detergent micelle contribution (Fig. 3C; Fig. S2B). DDM micelles were determined to be around 70 kDa without protein and increased in mass slightly when protein was incorporated. Two peaks eluted from the size exclusion column, and after micelle subtractions, the apparent masses of these proteins corresponded nicely with the hexamer (100 to 150 kDa) and the monomer (20 to 30 kDa) of TtFtsK_N_, which has a nominal mass of 23.2 kDa when monomeric. We conclude that the isolated N-terminal domain of FtsK, FtsK_N_, in solution has a clear, but not absolute, propensity to form hexamers and that the amount of hexamers formed depends on the amount of detergent present.

We do not know what regions of FtsK_N_ are required for hexamerization, although both the *E. coli* and *T. tengcongensis* proteins have short regions of C-terminal peptide (30 amino acids for *E. coli* FtsK_N_ used in the *in vivo* experiments, 24 amino acids for the *E. coli* FtsK protein used for biochemical characterization, and 10 amino acids for the *T. tengcongensis* protein) that are contained within the 50 amino acids shown to help functional oligomers of FtsK translocase to form *in vitro* (18). Nevertheless, it seems unlikely that these short peptides alone are sufficient for hexamerization.

### An *E. coli* cell contains 30 to ~100 molecules of FtsK.

**FIG 4:**
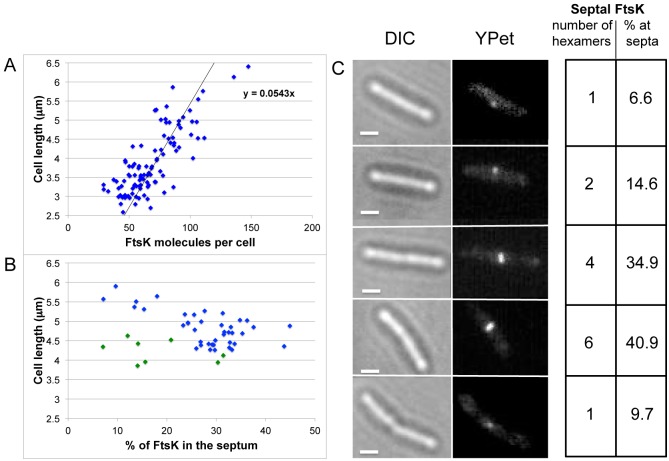
Numbers of FtsK molecules in cells. (A) Numbers of FtsK-YPet molecules as a function of cell length in a mixed population of dividing and nondividing cells. (B) Percentage of FtsK-YPet molecules that were localized at midcell in cells with a central FtsK-YPet signal as a function of cell length. Green symbols represent unconstricted cells. (C) Representative images of cells containing FtsK-YPet derivatives at different stages of the growth cycle. Differential interference contrast (DIC) and fluorescent images are displayed, and the number of FtsK hexamers, the cell length, and the percentage of molecules present at midcell are shown.

Given that up to 7 FtsK hexamers were present at midcell, it was important to determine what fraction of cellular FtsK was recruited to midcell prior to and during septation and what the total copy number of FtsK molecules per cell was irrespective of whether FtsK molecules were present within the divisome. We measured the integrated fluorescent signal within a rectangular area encompassing whole cells and then subtracted the contribution to the total fluorescent signal derived from cell auto-fluorescence and from camera noise. We then used this corrected value to determine the total number of FtsK-YPet molecules in cells (Text S1). In a steady-state population of wild-type cells, the number of FtsK-YPet molecules varied from ~30 in newborn cells to >100 in long, dividing cells (mean of 69 ± 21 molecules per cell, with an approximate linear relationship between cell length and total FtsK copy number) (Fig. 4A).

Using the same methodology, we then analyzed the total numbers of FtsK molecules in a subpopulation of cells containing fluorescent signals at midcell and measured the number of FtsK molecules at midcell, as well as the number of total FtsK molecules per cell. We found that the percentages of FtsK present at midcell varied from 6 to 30% in unconstricted cells (green symbols in Fig. 4B) and up to ~45% in constricted cells (blue symbols). The percentage of FtsK molecules at midcell decreased in long, constricted cells, showing that FtsK is released from the septum during the last stages of cell division (blue symbols). Representative images of FtsK-derived signals in cells of different septation states are shown (Fig. 4C).

To confirm the validity of the above-described methodology, we used a second independent assay in which >90% the initial fluorescence was bleached to enable visualization of single FtsK fluorophores outside the center of the cell. This was because at the earliest time points of movies of cells expressing FtsK-YPet, the fluorescent signals could not be unambiguously resolved into single fluorophores because of the relatively high FtsK copy number. In contrast, the few single LacY-YPet molecules present in cells were readily identifiable and their fluorescence was quantifiable. We therefore analyzed movies of FtsK-YPet cells after ~90% of the molecules had been bleached, when single molecules similar in appearance and mobility to LacY-YPet molecules became visible. The bleach rate of YPet *in vivo* was determined using LacY-YPet and was fitted to a single exponential curve (Fig. S1B). The FtsK-YPet copy number in cells lacking midcell-positioned FtsK molecules was derived from the distribution of measured stoichiometries by extrapolating to time zero, before bleaching (Fig. S1C). The same population of cells was analyzed by measuring the integrated fluorescence as described above; the results are reported in Fig. S1D (the stoichiometry values obtained by the two methods fell within the same range [Fig. S1C and S1D]). This makes us confident of the validity of both methods.

When we analyzed the fluorescence signals of FtsK-YPet molecules outside the cell center, we found that the intensities of the spots that the custom-written Matlab code could identify within unbleached images of cells corresponded to either one or a maximum of two YPet molecules, with no evidence of higher-intensity spots in >99% of cells. In <1% of cells, a single bright spot having the intensity of a hexamer was observed. These bright spots were most often at a pole, indicating that hexamers may occasionally remain associated with a pole after septation is complete (Fig. S3). The presence of spots outside the cell center having the intensity of two molecules is probably the consequence of the relatively high number of FtsK molecules present, leading to two molecules being present in a diffraction-limited spot. Our failure to find evidence for the presence of FtsK hexamers away from midcell is consistent with FtsK being present largely as monomers prior to recruitment to midcell.

To gain insights into the movement of FtsK within the cell membrane, we determined the diffusion rate of FtsK outside the cell center and compared it with the diffusion of midcell FtsK. Single noncentral FtsK-YPet molecules were tracked over time and space in cells in which most of the fluorescent signal had been bleached, thereby allowing single FtsK fluorophores to be tracked. The mean square displacement [MSD(*r*^2^)] was calculated and plotted as a function of time. The same analysis was performed for single LacY-YPet molecules. The apparent diffusion coefficients of FtsK and LacY in the membrane were determined from the slope of the linear part of the curve and found to be similar, 0.039 µm^2^/s and 0.061 µm^2^/s, respectively (Fig. S4A and S4B, dark red and dark blue dots). These values are about 10-fold higher than the apparent diffusion coefficient of *E. coli* FliG, a protein which is part of the flagellar motor and which was found to diffuse in the cytoplasmic membrane as part of the basal body before the assembly of a fully functional flagellum (*D* = 0.005 μm^2^/s) (39). The observation that the diffusion coefficient of FtsK molecules outside the center was similar to that of monomeric LacY and higher than that of FliG, which diffuses in the cytoplasm within a very large complex, is also consistent with noncentral FtsK molecules being monomeric. The curves approached a horizontal asymptote at longer times, indicative of subdiffusive diffusion (40). The apparent diffusion coefficient of FtsK at midcell was also calculated, again after sufficient bleaching allowed the tracking of single fluorophores. FtsK at midcell was found to be virtually immobile (<0.0004 µm^2^/s), consistent with it being tethered to the divisome complex (Fig. S4A and S4B, green symbols).

In conclusion, just as the brightness analysis showed no evidence of proteins other than a rare FtsK hexamer outside the cell center, the diffusion analysis using either a 10-ms or 1-ms capture time gave no indication of a protein other than a single majority FtsK molecular species in the noncentral population, with a diffusion coefficient similar to that of LacY-YPet (Fig. S4B; compare the dark red and pink symbols for FtsK-YPet diffusion outside midcell and dark blue and light blue symbols for LacY-YPet diffusion).

### Stoichiometries of FtsQ, ZapC, and TolQ division proteins at midcell.

Since our data showed that the membrane-spanning N-terminal domain of FtsK hexamerized at midcell, it was important to assess the stoichiometries of other divisome proteins after they are recruited to the divisome. We chose FtsQ, whose recruitment to the divisome is FtsK dependent (17, 35), ZapC, and TolQ. Cytoplasmic ZapC is recruited to the divisome early in divisome assembly, while TolQ is an integral membrane protein that is recruited at the very end of division (41, 42).

**FIG 5:**
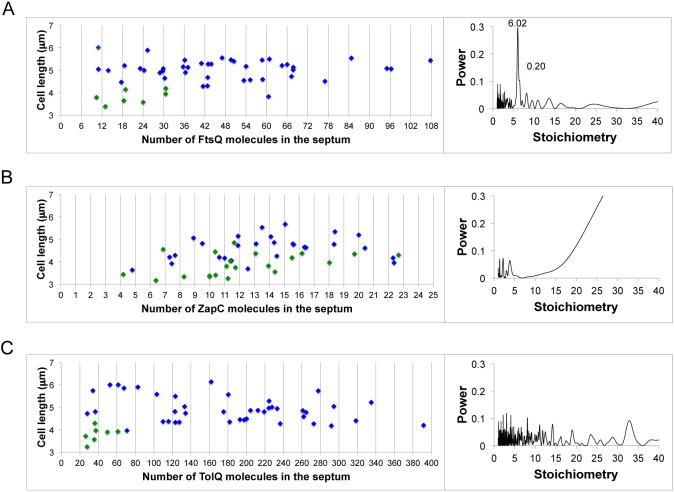
Stoichiometries of FtsQ, ZapC, and TolQ at midcell. (A, B, and C) Number of FtsQ, ZapC, and TolQ molecules, respectively, present at midcell as a function of cell length. Green symbols represent unconstricted cells, and blue symbols represent constricting cells. Power spectra on the right show periodicities, as in Fig. 2.

Like FtsK, FtsQ was present as multiples of 6, with a midcell copy number ~2-fold higher than the cellular copy number determined by alkaline phosphatase activity of a fusion protein (Fig. 5A) (43). FtsQ interacts with a range of upstream and downstream division proteins through both its small cytoplasmic domain and its periplasmic domain (44). Since FtsK is required to recruit FtsQ to the divisome, it seems possible that the observed hexameric clusters of FtsQ arise as a consequence of the interaction of FtsQ with FtsK_N_ (15, 44–46). In contrast, neither ZapC nor TolQ showed any evidence of being present as hexamers, or indeed as any other discrete oligomeric form, although we note that TolQ dimers were identified by *in vivo* cross-linking studies (47) (Fig. 5B and C). These data provide additional confirmation that the methodology used does not artifactually “find” hexamers.

### Concluding remarks.

We have shown that FtsK is present as hexamers at midcell and that the FtsK N-terminal domain is sufficient for this hexamerization. The methodologies used should be generally applicable to studies of divisome assembly and architecture. Our demonstration of *in vivo* hexamers is supported by the *in vitro* protein chemistry experiments that show that FtsK_N_ can be isolated as hexamers *in vitro* (Fig. 3).

FtsK hexamers accumulate before visible cell constriction, immediately after recruitment to midcell. The presence of FtsK hexamers throughout cell division provides an efficient way for the cell to ensure that the FtsK C-terminal translocase can readily be assembled and be functional as soon as required for chromosome dimer resolution and/or chromosome segregation. Are there other functional reasons for hexamerization of FtsK_N_? Studies of the FtsK ortholog SpoIIIE have led to the proposal that this protein translocates chromosomal DNA through a membrane pore formed by the N-terminal domain of SpoIIIE (48). The hexamerization of FtsK_N_ may provide such a pore, although we are not aware of any direct evidence to support the view that in *E. coli* a pore formed by FtsK_N_ is used for chromosomal DNA translocation (discussed in reference 34).

## MATERIALS AND METHODS

All strains used in this work are derivatives of *E. coli* K-12 AB1157. Standard genetic techniques were used for strain construction and protein purification and are described in detail in Text S1 in the supplemental material. Microscopy was performed using an OMX V3 Blaze microscope (GE Healthcare, Issaquah, WA) in widefield mode with a pco.edge sCMOS camera and the provided acquisition software. Image analysis was performed used a custom-written Matlab code. Details for image analysis and diffusion coefficient calculation are also presented in Text S1 in the supplemental material.

## SUPPLEMENTAL MATERIAL

Text S1 Supplemental materials and methods. Download Text S1, DOCX file, 0.1 MB

Figure S1 Calculation of the number of FtsK molecules present at midcell and in whole cells. (A) Auto-fluorescence of wild-type cells as a function of cell length. (B) Plot of the fraction of LacY-YPet fluorophores remaining as a function of time during bleaching. Movies of 477 LacY-YPet molecules were analyzed. The red line shows the single exponential fit. Each frame was 0.0175 s. (C) Number of FtsK molecules in nondividing cells calculated by “bleaching analysis.” A histogram representing the distribution of FtsK copy number observed after bleaching of 92.2% of the initial signal is reported, and the corresponding extrapolated copy number prior to bleaching and mean cell length values are displayed. (D) Numbers of FtsK molecules in nondividing cells as a function of cell length, calculated by “snapshot analysis,” plotted against cell length. The best linearly fitted line is shown. Download Figure S1, PDF file, 0.7 MB

Figure S2 (A) Multiple-sequence alignment of *E. coli*, *Thermoanaerobacter tengcongensis* (TtFtsK_N_), and 8 other FtsK homologues to show overall sequence conservation in the N-terminal domain, studied here. The alignment was calculated with T-COFFEE using default parameters. FtsK sequences used were *Pseudomonas aeruginosa*, *Bacillus subtilis*, *Chlorobium tepidum*, *Azospirillum brasilense*, *Xylella fastidiosa*, *Bradyrhizobium japonicum*, *Listeria monocytogenes*, and *Sporosarcina ureae*. (B) SEC-MALS of TtFtsK_N_ and DDM detergent, showing the same experiment as Fig. 3C. For clarity reasons, the traces resulting from injection of pure detergent were omitted from Fig. 3C and are shown here for completeness. The DDM detergent forms ~70-kDa micelles under the conditions used. Download Figure S2, PDF file, 2.2 MB

Figure S3 Noncentral polar FtsK hexamers. DIC and fluorescent images are displayed, and the number of FtsK hexamers is indicated. Download Figure S3, PDF file, 0.4 MB

Figure S4 Diffusion of FtsK-YPet and LacY-YPet. (A) Mean-square displacement [MSD(*r*^2^)] as a function of time for noncentral FtsK-YPet single fluorophores (red symbols), LacY-YPet single fluorophores (blue symbols), and FtsK-YPet single fluorophores present in hexamers at midcell (green symbols). The black lines are the linearly fitted over the first four data points in the case of FtsK-YPet and LacY-YPet located outside the cell center and over the whole data set in the case of FtsK-YPet at midcell. (B) Diffusion coefficients of FtsK-YPet molecules outside the cell center (red and pink symbols), LacY-YPet molecules (dark blue and light blue symbols), and FtsK-YPet molecules at midcell (green symbol) as measured by single-particle tracking. The mean value and the standard deviation for 30 independent measurements are displayed. Diffusion coefficient values represented by red, dark blue, and green symbols were obtained by acquiring movies at 10-ms exposure times and 10% laser power, while values represented by pink and light blue symbols were obtained from movies acquired using 1-ms exposure times and 100% laser power. Download Figure S4, PDF file, 0.7 MB

Table S1 *E. coli* K-12 AB1157 strains used in this work. Table S1, DOCX file, 0.1 MB.

Table S2 Oligonucleotides used in this work. Table S2, DOCX file, 0.1 MB.
