# Supplementary material for: The N-Terminal Membrane-Spanning Domain of the Escherichia coli DNA Translocase FtsK Hexamerizes at Midcell
Source: mBio. 2013 Dec 3;4(6):e00800-13. doi: 10.1128/mBio.00800-13 (PMC3870252; doi:10.1128/mBio.00800-13)
Supplement: Text S1 — Supplemental materials and methods. Download [file mbo006131685s1.docx]

**SUPPLEMENTAL MATERIAL**

**Title: The N-terminal membrane-spanning domain of the *Escherichia coli* DNA translocase FtsK hexamerizes at midcell**

**Authors**: Paola Bisicchia^a^, Bradley Steel^b,^ Mekdes H. Mariam Debela^c^, Jan Löwe^c^ and David Sherratt^a,1^

**Affiliations**: Departments of ^a^Biochemistry and ^b^Physics, University of Oxford, South Parks Road, Oxford OX13QU, UK.

^c^MRC Laboratory of Molecular Biology, Francis Crick Avenue, Cambridge Biomedical Campus, Cambridge CB2 0QH, UK

^1^To whom correspondence should be addressed.

Email: david.sherratt@bioch.ox.ac.uk. Telephone: +44(0)1865613237. Fax: +44(0)1865613238

**Running Title**: hexameric F­­­tsK at midcell

**Keywords:** bacterial cell division, divisome, FtsK, stoichiometry

**SUPPLEMENTAL MATERIALS AND METHODS**

**Construction of fluorescent fusions strains**

All strains used in this work are derivatives of *E. coli* K12 AB1157 (1) and are listed in Table1. The sequences of the oligonucleotides used in the construction of strains are listed in Table 2. Strains carrying fluorescent fusions of proteins to YPet were constructed by λ-Red recombination as previously described (2). For C-terminal and N-terminal fusion proteins, plasmids pRod61 and pRod44 were used respectively as templates for PCR amplification of genes coding for YPet and the Kanamycin resistance cassette (2). The sequence of the plasmids is reported below. Strain PB85, containing a C-terminal fluorescent fusion of FtsK to YPet, was constructed by λ-Red recombination (2). Strain PB355, containing a LacY-YPet derivative, was constructed by P1 transduction using strain RRL528 as a donor strain, selecting for kanamycin resistance. Strain RRL528, a kind gift from Dr Rodrigo Reyes-Lamothe, was constructed by λ-Red recombination in an MG1655 genetic background. Oligonucleotides used to construct *ftsK-yPet* were FtsK-F and FtsK-R, while primers used to construct *lacY-yPet* were LacY-F and LacY-R. The fraction of dark, immature YPet fluorophores in cells containing YPet fluorescent derivatives was previous found to be negligible (2). Strains PB107, PB166 and PB178, containing YPet-FtsQ, ZapC-YPet, TolQ-YPet fluorescent derivatives were constructed by λ-Red recombination using oligonucleotides FtsQ-F and FtsQ –R, ZapC-F and ZapC-R, TolQ-F and TolQ-R. In these strains, the *frt-*flanked kanamycin resistance cassette was removed by expressing Flp recombinase in order to prevent changes in expression caused by the *kan* promoter*,* leaving an *frt* scar immediately upstream of the initiation codon of *yPet* in the case of *yPet-ftsQ,* and immediately downstream of the stop codon of *yPet* in the case of *zapC-yPet* and *tolQ-yPet* (2). Strains expressing fusion proteins grew with normal length and division characteristics, and did not display any noticeable cell growth and morphology defects. In addition, the fusion protein correctly localized to septa at the expected time within the cell cycle, indicating that the presence of the fluorophore did not affect its function

Strain PB356, carrying *ftsK*Δ*_C_-yPet* (Fig. 2B) was made by first generating a deletion mutation in the C-terminal motor domain of FtsK by insertion-excision, resulting in a truncated variant of FtsK ending at amino acid 818, and then by generating a fluorescent derivative of this by λ−Red recombination as described above, using the oligonucleotide pair FtsK-F and FtsK-R-cm. Strain PB391, carrying a *ftsK*_N_*-yPet*, was derived from strain FX96, which contains a FtsK variant lacking the linker and the C-terminal translocase domain and encodes aa 1-210 (3), by fusing the truncated protein to YPet through λ-Red recombination, using the oligonucleotide pair FtsKFX96-F and FtsKFX96-R.

**Preparation of cells for microscopy**

Cells were grown at 37°C with shaking in LB until early exponential phase and then subcultured overnight in M9 glycerol. The following day, cultures with an A_600_ between 0.1 and 0.4 were diluted to an A_600_ of 0.02 and grown until A_600_ 0.1. Cells were then concentrated and laid on a M9-glycerol 1% agarose pad (4).

**Image acquisition**

We used an OMX V3 BLAZE microscope, (GE Healthcare, Issaquah, USA) in widefield mode. Images were acquired using a pico.edge sCMOS camera and the provided acquisition software. For each field of view (512x512 pixels

equal to 42x42 μm, in size), transmission images were acquired to

identify cell contours, as well as fluorescent images using a 514 nm laser

for excitation with emission detected through a 550/49 nm filter. Movies

of 400 frames were acquired with capture times of 10 ms, and with 7.5 ms

intervals between frames, and laser power at 10% of its maximum. Some

experiments were also performed with 1ms capture times, and with 7.5 ms

intervals between frames and 100% laser power.

**Image analysis: spot detection**

In order to define fluorescent spots within cells, we analyzed cell images with a custom written Matlab code. In order to determine the microscope PSF, we prepared a sample of 20 nm fluorescent beads mixed with a sparse surface coverage of cells. The cells were used to determine the focal plane in brightfield mode, then fluorescence images were collected for 500 frames. Beads located on the edge of the image, within 0.65 µm of another bead, or with any pixels saturated, were ignored. All other beads were fit using a circularly symmetric Gaussian of variable width. This selection was further limited by removing beads fit with a peak of less than 150 counts (below around 100 counts, noise from the background contributed to, on average, larger fit PSFs). This left a total of 4073 estimated PSF widths, and the median of these (fit standard deviation of 1.3 pixels) was used as the microscope PSF in future fits. In order to identify fluorescent spots within cells, we removed the cell autofluorescence signal, which gave a high, non-uniform background that was not associated with YPet. The image background was estimated by running a 2-d median filter, with a 5x5 window, over each image ten times sequentially. This filter was effective in removing spots of size similar to a PSF, but did not remove the larger patterns due to cell autofluorescence. Multiple overlapping Gaussian spots (5) were fit to the image after subtraction of the estimated background, using the fixed PSF measured previously. This process was then iterated by numerically subtracting detected Gaussians from the original image prior to the median filtering step, to remove any residual trace of real spots. In total four Gaussian fits were performed; further iterations had a negligible effect on the calculated background or amplitude of the spots.

We used the Matlab code supplied by Jaqaman (5) for multivariate Gaussian fitting, modified to assume uniform background noise properties, with a spot detection threshold equivalent to 5.5 standard deviations in the noise background, and with the minimum necessary code alterations required to run stably on our images. When required, we used tracking software from the same paper to convert spots detected in different video images, into tracks that spanned multiple images. While spots were identified in an automated way, cell contours were identified manually using the DIC images as a reference.

**Single fluorophore calibration**

In order to measure the intensity of single YPet fluorofore, we used strain PB355, which carries a LacY-YPet fluorescent derivative expressed from its native promoter. A similar construct was studied by Choi *et al* (6) and found to express between 0 and 10 LacY molecules per cell in the absence of any inducer. Movies of fluorescent images were analyzed using the Matlab code described above and cells displayed between 0 and 10 fluorescent spots, as previously observed (6). Tracks of fluorescent spots displayed single-step bleaching, indicating that each spot contained a single YPet fluorophore. In addition, the initial intensities of such tracks formed a uni-modal distribution, confirming that all analysed spots were single fluorophores. The initial intensities of 85 of such tracks were averaged and the resulting value (29.96 fluorescent units with a standard deviation of 2.09 fluorescent units) was then used to estimate the brightness of a single fluorophore.

**Determination of FtsK stoichiometry at midcell**

Cells expressing a FtsK-YPet construct displayed a patchy fluorescent signal outside of the cell centre, as well as an elongated brighter signal at midcell. We reasoned that a high number of FtsK molecules outside of the cell centre might prevent identification of single fluorophores and result in the observed patchy pattern. Because the number of spots that the Matlab code is capable of identifying per cell is limited, we could not use this software to measure the cellular stoichiometry of FtsK outside of midcell. However, we were able to determine the number of FtsK molecules at midcell using a modified version of the software. Fluorescent signals from midcell were noticeably stretched in one axis, and hence could not be accurately fit using a fixed-width PSF. We therefore used the position of the fixed-width PSF fit to seed a 6-parameter (elliptical) Gaussian fit to the first background subtracted image of a video, with two fit parameters for position, and one each for orientation and amplitude. We then used the integrated signal within the Gaussian fit, divided by the corresponding signal level from a single fluorophore (29.96), as a measure of the number of fluorophores present at midcell. A small correction (typically <3%) was applied to offset the estimated loss of fluorescence due to bleaching during image acquisition. The magnitude was based on an exponential fit of the bleach rate from multiple image frames in collected videos, and was not used for single fluorophores where bleaching shows up as a complete loss of signal. The multiplicative correction used, for a bleach time constant τ, was:

$$\frac{1}{\tau\left( 1-e^{-1/\tau} \right)}$$

**Analysis of the periodicity of FtsK stoichiometry at midcell**

In order to highlight any periodicities in the measured brightness values of FtsK at midcell, we generated a power spectrum of the measured stoichiometries, *s*, using:

$\text{PS}\left( f \right)=\left| \int{p(s)e}^{-i2\pi fs}ds \right|^{2}=\left| \sum e^{-i2\pi fs} \right|^{2}$

This power spectrum is plotted in Fig. 2 in inverse frequency space (1/*f*) such that the position of a peak corresponds to the associated stoichiometry.

**Determination of FtsK stoichiometry in cells**

The number of FtsK-YPet molecules per cell was calculated using Fiji by measuring the integrated fluorescence of a rectangular area encompassing the whole cell and then subtracting the contribution to the total fluorescence coming from the cell auto-fluorescence and from the camera noise. When analyzing the integrated fluorescence of 100 wild-type cells, it was found that although there is some increase of cell auto-fluorescence with cell length, the correlation was not strong (Fig. S1A). Consequently, we used the mean auto-fluorescence (2938 fluorescent units) and subtracted this from the total integrated fluorescence for all cells, independently of cell length. The standard deviation from the mean for cell auto-fluorescence was 1309.9 fluorescent units, which reflects the error coming from the natural variation in cell auto-fluorescence. The contribution to the total fluorescence coming from the camera noise was calculated by measuring the integrated fluorescence over a rectangle of the same dimensions adjacent to the cell and devoid of cells. The error coming from the camera noise was calculated by measuring the difference in fluorescence values between two adjacent rectangles aligned vertically in a field. This measurement was repeated 100 times and the mean value (50.57 fluorescent units) and standard deviation (846.97 units) was calculated. The mean value was close to zero, as expected, and the standard deviation is a reflection of the error in the stoichiometry calculation coming from the camera noise variation. In order to calculate the total number of FtsK-YPet molecules in cells, the integrated fluorescence value after subtraction of the autofluorescence and camera noise contributions was divided by the integral area under a spot of central brightness 1 (10.62), which allows one to convert fluorescence values obtained with Fiji analysis to fluorescence values used in the Matlab software. The converted value was further divided by 29.96, which corresponds to the intensity of a single fluorophore, therefore determining the stoichiometry of FtsK in whole cells. The standard deviation of the mean cell auto-fluorescence and of the camera noise (1309.9 and 846.97 in Fiji fluorescent units, respectively), when converted into the Matlab software value and then divided by the intensity of a single YPet molecule, correspond to 4.12 and 2.66 YPet fluorophores respectively. Therefore an error of plus or minus 6 molecules is expected when calculating the stoichiometry of FtsK with Fiji.

To validate the data from the above method, we adopted an alternative method for calculating the stoichiometry of FtsK in cells. Briefly, we calculated the number of FtsK-YPet molecules present in whole cells in the first frame of movies by counting the number of fluorescent spots corresponding to a single fluorophore after bleaching of most of the signal, and by knowing the bleaching rate of YPet fluorofores. First, we calculated the bleaching rate of YPet by analyzing movies of cells expressing LacY-YPet derivatives and measuring the number of frames required for bleaching of single fluorophores. We analyzed a total of 477 LacY-YPet molecules, and plotted the fraction of total molecules surviving bleaching as a function of time (Fig. S1B). We then analyzed movies of cells expressing FtsK-YPet derivatives, and counted the number of fluorescent spots identified by the Matlab software after bleaching of most of the fluorescent signal. We chose to count the number of fluorescent spots visible at frame 35, when 7.8% of the initial fluorescence remained and isolated FtsK-YPet fluorescent spots, very similar in appearance and mobility to LacY-YPet molecules, were visible and identified as single spots by the Matlab Software, which is unable to identify more than 10-15 spots. We calculated the number of FtsK molecules present in whole cells by dividing the number of fluorescent spots visible at frame 35 by the fraction of residual LacY-YPet molecules present at this stage (Fig. S1C). As a control, we also calculated the decay rate of YPet by fitting an exponential trendline to the histogram in Fig. S1B. The corresponding formula was:

$\text{N}\left( n \right)={0.92702 N\left( 0 \right)*e}^{-0.07223 n}$

Where N(n) is the number of fluorophores present in a cell at frame number n, and N(0) is the number of fluorophores present in the initial frame. When applying this formula to calculate the number of initial FtsK-YPet molecules from the number of spots visible at frame 35, we obtained values that were only 4.6% different from the ones obtained with the previous method, confirming the validity of our approach.

We then compared the two different methods used to determine the number of FtsK molecules in whole cells by using them to calculate the total number of FtsK molecules in the same population of non-dividing cells (Fig. S1D). We found that the stoichiometry values determined by the two methods gave comparable results (Fig. S1C and S1D).

**Determination of FtsQ, ZapC and TolQ stoichiometries at midcell**

The number of FtsQ, ZapC and TolQ proteins present at midcell was determined as described for FtsK.

**Determination of FtsK stoichiometries at midcell after bleaching**

In order to validate the methodology used for determining the number of cell division molecules at midcell, we analyzed the same movies utilized for measuring the number of FtsK at midcell, after subtracting the first 10 frames. This results bleaching of the fluorescent signal deriving from FtsK-YPet to about 50% of its initial value (see Fig. S1B). We then determined the stoichiometry of FtsK at midcell as outlined above.

**Diffusion coefficients for FtsK-YPet and LacY-YPet**

The diffusion coefficient of FtsK-YPet molecules located outside of midcell and LacY-YPet molecules were measured using the Fiji plugin 5D viewer for single particle tracking. While LacY-YPet single molecules were easily identifiable in fluorescent movies from frame 1, the fluorescent signals in cells containing FtsK-YPet derivatives were initially patchy, due to the high number of FtsK molecules in cells. Therefore we used frames 100 to 400, so that most of the initial signal was bleached, leaving clearly distinguishable single FtsK-YPet particles. Single fluorescent molecules were tracked over time and space, and the mean square displacement [MSD(r^2^)] was calculated as described in (7) and plotted as a function of time. Representative graphs are shown in Fig. S4A The shape of the MSD curve versus time was used to classify trajectories (7). Both in the case of LacY-YPet outside the cell centre and of FtsK-YPet, the MSD curves approach an horizontal asymptote, typical of sub-diffusive motion. The diffusion coefficient D was calculating by fitting a linear line over the first 4 data points of the MSD versus time curve, and using the slope of such line according to the equation:

$MSD(r2) ={4Dt}^{a}$

The mean value and standard deviation for 30 independent measurements was calculated for both non-central LacY-YPet and FtsK-YPet molecules. Images were acquired with 10 ms exposure times and 10% laser power or with 1ms exposure time and 100% laser power.

The diffusion coefficient of FtsK-YPet molecules located at midcell was also calculated as described above, using 10 ms exposure times. Movies of dividing cells were analyzed after most of the initial fluorescence had bleached, in order to ensure that the FtsK hexamers were represented by a single fluorofore. We verified that the brightness of such spots corresponded to that of a single YPet fluorophore by measuring the intensity of the spots with the custom Matlab code described above. The shape of the MSD versus time curve indicated normal diffusion, and the diffusion coefficient was calculated by fitting a linear line over the whole data set and using the slope of such line according to the formula described above.

**Cloning, expression and purification of EcFtsK and TtFtsK N-terminal domains (EcFtsK_N_, TtFtsK_N_)**

The DNA fragment encoding residues 2 to 190 of *Thermoanaerobacter tengcongensis* FtsK (transmembrane domain only, NCBI reference NP_622996) and a C-terminal 10 histidine-tag was cloned into T7 expression plasmid pHis17 (B. Miroux, MRC-LMB) using NdeI/BamHI restriction sites and overexpression was performed in C43(DE3) cells. Using the same methods and tag, residues 1-204 of *Escherichia coli* FtsK (UNIPROT ID FTSK_ECOLI) were cloned into pHis17 and expressed in C43(DE3) cells. Cells were grown in LB medium at 37°C and induced at OD 0.3 / 22°C with 0.4 mM isopropyl-ß-thiogalactoside (IPTG) for 5 hours. Cells were harvested by centrifugation at 10,000x g, resuspended in PBS, EDTA-free protease inhibitor cocktail (Roche), RNase A (Sigma), DNase 1 (Sigma) and then lysed with a cell disruptor (Constant System) at 35 KPSI. Debris was removed by centrifugation at 10,000x g. The supernatant was collected and centrifuged at 200,000x g for 1.5 h to obtain a clear membrane pellet. The pelleted membranes were homogenised in a Dounce homogeniser, and then solubilised in buffer containing 50 mM Tris/HCl, 300 mM NaCl, 10 mM imidazole, 10 % glycerol, 40 mM n-dodcyl β-D-maltopyranoside (DDM) (Glycon), adjusted to pH 8.0, at 4°C for an hour. Soluble material was isolated by centrifugation at 80,000x g for 30 min. The supernatant was loaded onto a 5 ml Talon cobalt metal affinity column (Clonetech). The loaded Talon resin was washed until the baseline was stable and eluted with a gradient of 300-500 mM imidazole in resuspension buffer. Fractions were checked for the eluted FtsK_N_ proteins by SDS-PAGE and at this stage show a single or double band. Eluted proteins were then concentrated in 100 kDa MWCO Vivaspin centrifugal concentrators (Sartorius) and further purified using a Superdex S-200 10/300 size-exclusion column (GE Healthcare) in buffer containing 50 mM Tris/HCl, 150 mM NaCl, 1 mM DDM and 10% glycerol, adjusted to pH 8.0. The proteins eluted as single peaks, corresponding approximately to the hexameric form of FtsK_N_. To verify identity and integrity of the purified FtsK_N_s, samples were prepared for electrospray ionization mass spectrometry analysis (ESMS): fractions from the Superdex S-200 column were treated with 60% v/v formic acid to strip off the bound detergent and lipid from the sample and centrifuged for 5 min in a microcentrifuge and the pellets were dissolved with 50% v/v methanol, 25% v/v acetonitrile and 5% v/v formic acid. Expected mass: 23218.4 Da, observed mass: 23220.0 Da for Tt.

Size exclusion chromatography with multi-angle light scattering (SEC-MALS)

TtFtsK_N_ was resolved on a Superdex S-200 10/300 SEC column (GE Healthcare) in 50 mM Tris/HCl, 100 mM NaCl, 10% w/v glycerol, pH 7.5 with 1 mM DDM and detected by UV at 280 nm (Agilent 1200 MWD), light scattering (Wyatt Heleos II) and refractive index (Wyatt Optilab rEX). 100 µl sample were injected at a concentration of 3 mg/ml. The masses of TtFtsK_N_ and DDM were determined using the dual detection method as implemented in Wyatt’s ASTRA analysis software as conjugate analysis (Wyatt Technology). The protein refractive index increment used was 0.186 ml g^-1^ and the extinction coefficient for UV detection at 280 nm was 2000 ml g^-1^ cm^-1^ for *TtFtsK_N_*. DDM refractive index increment used was 0.13 ml g^-1^ and the DDM extinction coefficient for UV detection at 280 nm used was 27 ml g^-1^ cm^-1^. The UV value was determined from control measurements of DDM injected from a concentrated stock solution, in which refractive index monitoring indicated a micelle mass of 66.5 kDa in agreement with literature values (8) (Fig. S2B). The UV signal during these measurements was independently used to analyse the micelle mass and the UV extinction coefficient adjusted until a mass consistent with the value determined by refractive index was obtained. The inter-detector delay volumes, and associated band broadening constants, as well as the detector intensity normalisation constants for the Heleos and the UV intensity calibration were determined prior to each set of measurements using known protein standards (IgG and BSA).

**SUPPLEMENTAL REFERENCES**

1. [Bachmann BJ](http://www.ncbi.nlm.nih.gov/pubmed?term=Bachmann%20BJ%5BAuthor%5D&cauthor=true&cauthor_uid=4568763). 1972. Pedigrees of some mutant strains of *Escherichia coli* K-12. Bacteriol. Rev. **36:** 525-557.

2. [Reyes-Lamothe R](http://www.ncbi.nlm.nih.gov/pubmed?term=Reyes-Lamothe%20R%5BAuthor%5D&cauthor=true&cauthor_uid=20413500), [Sherratt DJ](http://www.ncbi.nlm.nih.gov/pubmed?term=Sherratt%20DJ%5BAuthor%5D&cauthor=true&cauthor_uid=20413500), [Leake MC](http://www.ncbi.nlm.nih.gov/pubmed?term=Leake%20MC%5BAuthor%5D&cauthor=true&cauthor_uid=20413500). 2010. Stoichiometry and architecture of active DNA replication machinery in *Escherichia coli*. Science **328:**498-501.

3. [Bigot S](http://www.ncbi.nlm.nih.gov/pubmed?term=Bigot%20S%5BAuthor%5D&cauthor=true&cauthor_uid=15522074), [Corre J](http://www.ncbi.nlm.nih.gov/pubmed?term=Corre%20J%5BAuthor%5D&cauthor=true&cauthor_uid=15522074), [Louarn JM](http://www.ncbi.nlm.nih.gov/pubmed?term=Louarn%20JM%5BAuthor%5D&cauthor=true&cauthor_uid=15522074), [Cornet F](http://www.ncbi.nlm.nih.gov/pubmed?term=Cornet%20F%5BAuthor%5D&cauthor=true&cauthor_uid=15522074), [Barre FX](http://www.ncbi.nlm.nih.gov/pubmed?term=Barre%20FX%5BAuthor%5D&cauthor=true&cauthor_uid=15522074). 2004. FtsK activities in Xer recombination, DNA mobilization and cell division involve overlapping and separate domains of the protein. Mol. Microbiol*.* **54:**876-86.

4. [Wang X](http://www.ncbi.nlm.nih.gov/pubmed?term=Wang%20X%5BAuthor%5D&cauthor=true&cauthor_uid=16204186), [Possoz C](http://www.ncbi.nlm.nih.gov/pubmed?term=Possoz%20C%5BAuthor%5D&cauthor=true&cauthor_uid=16204186), [Sherratt DJ](http://www.ncbi.nlm.nih.gov/pubmed?term=Sherratt%20DJ%5BAuthor%5D&cauthor=true&cauthor_uid=16204186) (2005) Dancing around the divisome: asymmetric chromosome segregation in *Escherichia coli.* Genes. Dev. **19:**2367-2377.

5. [Jaqaman K](http://www.ncbi.nlm.nih.gov/pubmed?term=Jaqaman%20K%5BAuthor%5D&cauthor=true&cauthor_uid=18641657), [Loerke D](http://www.ncbi.nlm.nih.gov/pubmed?term=Loerke%20D%5BAuthor%5D&cauthor=true&cauthor_uid=18641657), [Mettlen M](http://www.ncbi.nlm.nih.gov/pubmed?term=Mettlen%20M%5BAuthor%5D&cauthor=true&cauthor_uid=18641657), [Kuwata H](http://www.ncbi.nlm.nih.gov/pubmed?term=Kuwata%20H%5BAuthor%5D&cauthor=true&cauthor_uid=18641657), [Grinstein S](http://www.ncbi.nlm.nih.gov/pubmed?term=Grinstein%20S%5BAuthor%5D&cauthor=true&cauthor_uid=18641657), [Schmid SL](http://www.ncbi.nlm.nih.gov/pubmed?term=Schmid%20SL%5BAuthor%5D&cauthor=true&cauthor_uid=18641657), [Danuser G](http://www.ncbi.nlm.nih.gov/pubmed?term=Danuser%20G%5BAuthor%5D&cauthor=true&cauthor_uid=18641657). 2008. Robust single-particle tracking in live-cell time-lapse sequences. Nat. Methods. **5:**695-702.

6. [Choi PJ](http://www.ncbi.nlm.nih.gov/pubmed?term=Choi%20PJ%5BAuthor%5D&cauthor=true&cauthor_uid=18927393), [Cai L](http://www.ncbi.nlm.nih.gov/pubmed?term=Cai%20L%5BAuthor%5D&cauthor=true&cauthor_uid=18927393), [Frieda K](http://www.ncbi.nlm.nih.gov/pubmed?term=Frieda%20K%5BAuthor%5D&cauthor=true&cauthor_uid=18927393), [Xie XS](http://www.ncbi.nlm.nih.gov/pubmed?term=Xie%20XS%5BAuthor%5D&cauthor=true&cauthor_uid=18927393). 2008. A stochastic single-molecule event triggers phenotype switching of a bacterial cell. Science **322:**442-446.

7. [Saxton MJ](http://www.ncbi.nlm.nih.gov/pubmed?term=Saxton%20MJ%5BAuthor%5D&cauthor=true&cauthor_uid=9241424), [Jacobson K](http://www.ncbi.nlm.nih.gov/pubmed?term=Jacobson%20K%5BAuthor%5D&cauthor=true&cauthor_uid=9241424). 1997. Single-particle tracking: applications to membrane dynamics. Annu. Rev. Biophys. Biomol. Struct. **26:**373-399.

8. Slotboom DJ, Duurkens RH, Olieman K, Erkensa GB. 2008. Static light scattering to characterize membrane proteins in detergent solution. Methods **46:**73-82.

**SEQUENCES OF PLASMIDS USED IN THIS WORK**

**pROD61 (YPet- Kan orig R6K)**

GACGAAAGGGCCTCGTGATACGCCTATTTTTATAGGTTAATGTCATGATAATAATGGTTTCTTAGACGTCCCATGGCTAATTCCCATGTCAGCCGTTAAGTGTTCCTGTGTCACTGAAAATTGCTTTGAGAGGCTCTAAGGGCTTCTCAGTGCGTTACATCCCTGGCTTGTTGTCCACAACCGTTAAACCTTAAAAGCTTTAAAAGCCTTATATATTCTTTTTTTTCTTATAAAACTTAAAACCTTAGAGGCTATTTAAGTTGCTGATTTATATTAATTTTATTGTTCAAACATGAGAGCTTAGTACGTGAAACATGAGAGCTTAGTACGTTAGCCATGAGAGCTTAGTACGTTAGCCATGAGGGTTTAGTTCGTTAAACATGAGAGCTTAGTACGTTAAACATGAGAGCTTAGTACGTGAAACATGAGAGCTTAGTACGTACTATCAACAGGTTGAACTGCGGATCTTGACATGTTCTTTCCTGCGTTATCCCCTGATTCTGTGGATAACCGTATTACCGCCTTTGAGTGAGCTGATACCGCTCGCCGCAGCCGAACGACCGAGCGCAGCGAGTCAGTGAGCGAGGAAGCGGAAGAGCGCCCAATACGCAAACCGCCTCTCCCCGCGCGTTGGCCGATTCATTAATGCAGCTGGCACGACAGGTTTCCCGACTGGAAAGCGGGCAGTGAGCGCAACGCAATTAATGTGAGTTAGCTCACTCATTAGGCACCCCAGGCTTTACACTTTATGCTTCCGGCTCGTATGTTGTGTGGAATTGTGAGCGGATAACAATTTCACACAGGAAACAGCTATGACCATGATTACGAATTCGAGCTCGGCTGGCTCCGCTGCTGGTTCTGGCGAATTCGTGTCTAAAGGTGAAGAATTATTCACTGGTGTTGTCCCAATTTTGGTTGAATTAGATGGTGATGTTAATGGTCACAAATTTTCTGTCTCCGGTGAAGGTGAAGGTGATGCTACGTACGGTAAATTGACCTTAAAATTACTCTGTACTACTGGTAAATTGCCAGTTCCATGGCCAACCTTAGTCACTACTTTAGGTTATGGTGTTCAATGTTTTGCTAGATACCCAGATCATATGAAACAACATGACTTTTTCAAGTCTGCCATGCCAGAAGGTTATGTTCAAGAAAGAACTATTTTTTTCAAAGATGACGGTAACTACAAGACCAGAGCTGAAGTCAAGTTTGAAGGTGATACCTTAGTTAATAGAATCGAATTAAAAGGTATTGATTTTAAAGAAGATGGTAACATTTTAGGTCACAAATTGGAATACAACTATAACTCTCACAATGTTTACATCACTGCTGACAAACAAAAGAATGGTATCAAAGCTAACTTCAAAATTAGACACAACATTGAAGATGGTGGTGTTCAATTAGCTGACCATTATCAACAAAATACTCCAATTGGTGATGGTCCAGTCTTGTTACCAGACAACCATTACTTATCCTATCAATCTGCCTTATTCAAAGATCCAAACGAAAAGAGAGACCACATGGTCTTGTTAGAATTTTTGACTGCTGCTGGTATTACCGAGGGTATGAATGAATTGTACAAATAACCCGGGTGTAGGCTGGAGCTGCTTCGAAGTTCCTATACTTTCTAGAGAATAGGAACTTCGGAATAGGAACTTCAAGATCCCCTCACGCTGCCGCAAGCACTCAGGGCGCAAGGGCTGCTAAAGGAAGCGGAACACGTAGAAAGCCAGTCCGCAGAAACGGTGCTGACCCCGGATGAATGTCAGCTACTGGGCTATCTGGACAAGGGAAAACGCAAGCGCAAAGAGAAAGCAGGTAGCTTGCAGTGGGCTTACATGGCGATAGCTAGACTGGGCGGTTTTATGGACAGCAAGCGAACCGGAATTGCCAGCTGGGGCGCCCTCTGGTAAGGTTGGGAAGCCCTGCAAAGTAAACTGGATGGCTTTCTTGCCGCCAAGGATCTGATGGCGCAGGGGATCAAGATCTGATCAAGAGACAGGATGAGGATCGTTTCGCATGATTGAACAAGATGGATTGCACGCAGGTTCTCCGGCCGCTTGGGTGGAGAGGCTATTCGGCTATGACTGGGCACAACAGACAATCGGCTGCTCTGATGCCGCCGTGTTCCGGCTGTCAGCGCAGGGGCGCCCGGTTCTTTTTGTCAAGACCGACCTGTCCGGTGCCCTGAATGAACTGCAGGACGAGGCAGCGCGGCTATCGTGGCTGGCCACGACGGGCGTTCCTTGCGCAGCTGTGCTCGACGTTGTCACTGAAGCGGGAAGGGACTGGCTGCTATTGGGCGAAGTGCCGGGGCAGGATCTCCTGTCATCTCACCTTGCTCCTGCCGAGAAAGTATCCATCATGGCTGATGCAATGCGGCGGCTGCATACGCTTGATCCGGCTACCTGCCCATTCGACCACCAAGCGAAACATCGCATCGAGCGAGCACGTACTCGGATGGAAGCCGGTCTTGTCGATCAGGATGATCTGGACGAAGAGCATCAGGGGCTCGCGCCAGCCGAACTGTTCGCCAGGCTCAAGGCGCGCATGCCCGACGGCGAGGATCTCGTCGTGACCCATGGCGATGCCTGCTTGCCGAATATCATGGTGGAAAATGGCCGCTTTTCTGGATTCATCGACTGTGGCCGGCTGGGTGTGGCGGACCGCTATCAGGACATAGCGTTGGCTACCCGTGATATTGCTGAAGAGCTTGGCGGCGAATGGGCTGACCGCTTCCTCGTGCTTTACGGTATCGCCGCTCCCGATTCGCAGCGCATCGCCTTCTATCGCCTTCTTGACGAGTTCTTCTGAGCGGGACTCTGGGGTTCGAAATGACCGACCAAGCGACGCCCAACCTGCCATCACGAGATTTCGATTCCACCGCCGCCTTCTATGAAAGGTTGGGCTTCGGAATCGTTTTCCGGGACGCCGGCTGGATGATCCTCCAGCGCGGGGATCTCATGCTGGAGTTCTTCGCCCACCCCAGCTTCAAAAGCGCTCTGAAGTTCCTATACTTTCTAGAGAATAGGAACTTCGGAATAGGAACTAAGGAGGATATTCATATGGGATCCTCTAGAGTCGACCTGCAGGCATGCAAGCTTGGCACTGGCCGTCGTTTTACAACGTCGTGACTGGGAAAACCCTGGCGTTACCCAACTTAATCGCCTTGCAGCACATCCCCCTTTCGCCAGCTGGCGTAATAGCGAAGAGGCCCGCACCGATCGCCCTTCCCAACAGTTGCGCAGCCTGAATGGCGAATGGCGCCTGATGCGGTATTTTCTCCTTACGCATCTGTGCGGTATTTCACACCGCATATGGTGCACTCTCAGTACAATCTGCTCTGATGCCGCATAGTTAAGCCAGCCCCGACACCCGCCAACACCCGCTGACGCGCCCTGACGGGCTTGTCTGCTCCCGGCATCCGCTTACAGACAAGCTGTGACCGTCTCCGGGAGCTGCATGTGTCAGAGGTTTTCACCGTCATCACCGAAACGCGCGA

**pROD44 (Kan-YPet orig R6K)**

GACGAAAGGGCCTCGTGATACGCCTATTTTTATAGGTTAATGTCATGATAATAATGGTTTCTTAGACGTCAGGTGGCACTTTTCGGGGAAATGTGCGCGGAACCCCTATTTGTTTATTTTTCTAAATACATTCAAATATGTATCCGCTCATGAGACAATAACCCTGATAAATGCTTCAATAATATTGAAAAAGGAAGAGTATGAGTATTCAACATTTCCGTGTCGCCCTTATTCCCTTTTTTGCGGCATTTTGCCTTCCTGTTTTTGCTCACCCAGAAACGCTGGTGAAAGTAAAAGATGCTGAAGATCAGTTGGGTGCACGAGTGGGTTACATCGAACTGGATCTCAACAGCGGTAAGATCCTTGAGAGTTTTCGCCCCGAAGAACGTTTTCCAATGATGAGCACTTTTAAAGTTCTGCTATGTGGCGCGGTATTATCCCGTATTGACGCCGGGCAAGAGCAACTCGGTCGCCGCATACACTATTCTCAGAATGACTTGGTTGAGTACTCACCAGTCACAGAAAAGCATCTTACGGATGGCATGACAGTAAGAGAATTATGCAGTGCTGCCATAACCATGAGTGATAACACTGCGGCCAACTTACTTCTGACAACGATCGGAGGACCGAAGGAGCTAACCGCTTTTTTGCACAACATGGGGGATCATGTAACTCGCCTTGATCGTTGGGAACCGGAGCTGAATGAAGCCATACCAAACGACGAGCGTGACACCACGATGCCTGTAGCAATGGCAACAACGTTGCGCAAACTATTAACTGGCGAACTACTTACTCTAGCTTCCCGGCAACAATTAATAGACTGGATGGAGGCGGATAAAGTTGCAGGACCACTTCTGCGCTCGGCCCTTCCGGCTGGCTGGTTTATTGCTGATAAATCTGGAGCCGGTGAGCGTGGGTCTCGCGGTATCATTGCAGCACTGGGGCCAGATGGTAAGCCCTCCCGTATCGTAGTTATCTACACGACGGGGAGTCAGGCAACTATGGATGAACGAAATAGACAGATCGCTGAGATAGGTGCCTCACTGATTAAGCATTGGTAACTGTCAGACCAAGTTTACTCATATATACTTTAGATTGATTTAAAACTTCATTTTTAATTTAAAAGGATCTAGGTGAAGATCCTTTTTGATAATCTCATGACCAAAATCCCTTAACGTGAGTTTTCGTTCCACTGAGCGTCAGACCCCGTAGAAAAGATCAAAGGATCTTCTTGAGATCCTTTTTTTCTGCGCGTAATCTGCTGCTTGCAAACAAAAAAACCACCGCTACCAGCGGTGGTTTGTTTGCCGGATCAAGAGCTACCAACTCTTTTTCCGAAGGTAACTGGCTTCAGCAGAGCGCAGATACCAAATACTGTCCTTCTAGTGTAGCCGTAGTTAGGCCACCACTTCAAGAACTCTGTAGCACCGCCTACATACCTCGCTCTGCTAATCCTGTTACCAGTGGCTGCTGCCAGTGGCGATAAGTCGTGTCTTACCGGGTTGGACTCAAGACGATAGTTACCGGATAAGGCGCAGCGGTCGGGCTGAACGGGGGGTTCGTGCACACAGCCCAGCTTGGAGCGAACGACCTACACCGAACTGAGATACCTACAGCGTGAGCTATGAGAAAGCGCCACGCTTCCCGAAGGGAGAAAGGCGGACAGGTATCCGGTAAGCGGCAGGGTCGGAACAGGAGAGCGCACGAGGGAGCTTCCAGGGGGAAACGCCTGGTATCTTTATAGTCCTGTCGGGTTTCGCCACCTCTGACTTGAGCGTCGATTTTTGTGATGCTCGTCAGGGGGGCGGAGCCTATGGAAAAACGCCAGCAACGCGGCCTTTTTACGGTTCCTGGCCTTTTGCTGGCCTTTTGCTCACATGTTCTTTCCTGCGTTATCCCCTGATTCTGTGGATAACCGTATTACCGCCTTTGAGTGAGCTGATACCGCTCGCCGCAGCCGAACGACCGAGCGCAGCGAGTCAGTGAGCGAGGAAGCGGAAGAGCGCCCAATACGCAAACCGCCTCTCCCCGCGCGTTGGCCGATTCATTAATGCAGCTGGCACGACAGGTTTCCCGACTGGAAAGCGGGCAGTGAGCGCAACGCAATTAATGTGAGTTAGCTCACTCATTAGGCACCCCAGGCTTTACACTTTATGCTTCCGGCTCGTATGTTGTGTGGAATTGTGAGCGGATAACAATTTCACACAGGAAACAGCTATGACCATGATTACGAGCTCGCGCTGCCAGAACCAGCGGCGGAGCCTGCCGATTTGTACAATTCATTCATACCCTCGGTAATACCAGCAGCAGTCAAAAATTCTAACAAGACCATGTGGTCTCTCTTTTCGTTTGGATCTTTGAATAAGGCAGATTGATAGGATAAGTAATGGTTGTCTGGTAACAAGACTGGACCATCACCAATTGGAGTATTTTGTTGATAATGGTCAGCTAATTGAACACCACCATCTTCAATGTTGTGTCTAATTTTGAAGTTAGCTTTGATACCATTCTTTTGTTTGTCAGCAGTGATGTAAACATTGTGAGAGTTATAGTTGTATTCCAATTTGTGACCTAAAATGTTACCATCTTCTTTAAAATCAATACCTTTTAATTCGATTCTATTAACTAAGGTATCACCTTCAAACTTGACTTCAGCTCTGGTCTTGTAGTTACCGTCATCTTTGAAAAAAATAGTTCTTTCTTGAACATAACCTTCTGGCATGGCAGACTTGAAAAAGTCATGTTGTTTCATATGATCTGGGTATCTAGCAAAACATTGAACACCATAACCTAAAGTAGTGACTAAGGTTGGCCATGGAACTGGCAATTTACCAGTAGTACAGAGTAATTTTAAGGTCAATTTACCGTACGTAGCATCACCTTCACCTTCACCGGAGACAGAAAATTTGTGACCATTAACATCACCATCTAATTCAACCAAAATTGGGACAACACCAGTGAATAATTCTTCACCTTTAGACATCCCGGGCATATGAATATCCTCCTTAGTTCCTATTCCGAAGTTCCTATTCTCTAGAAAGTATAGGAACTTCAGAGCGCTTTTGAAGCTGGGGTGGGCGAAGAACTCCAGCATGAGATCCCCGCGCTGGAGGATCATCCAGCCGGCGTCCCGGAAAACGATTCCGAAGCCCAACCTTTCATAGAAGGCGGCGGTGGAATCGAAATCTCGTGATGGCAGGTTGGGCGTCGCTTGGTCGGTCATTTCGAACCCCAGAGTCCCGCTCAGAAGAACTCGTCAAGAAGGCGATAGAAGGCGATGCGCTGCGAATCGGGAGCGGCGATACCGTAAAGCACGAGGAAGCGGTCAGCCCATTCGCCGCCAAGCTCTTCAGCAATATCACGGGTAGCCAACGCTATGTCCTGATAGCGGTCCGCCACACCCAGCCGGCCACAGTCGATGAATCCAGAAAAGCGGCCATTTTCCACCATGATATTCGGCAAGCAGGCATCGCCATGGGTCACGACGAGATCCTCGCCGTCGGGCATGCGCGCCTTGAGCCTGGCGAACAGTTCGGCTGGCGCGAGCCCCTGATGCTCTTCGTCCAGATCATCCTGATCGACAAGACCGGCTTCCATCCGAGTACGTGCTCGCTCGATGCGATGTTTCGCTTGGTGGTCGAATGGGCAGGTAGCCGGATCAAGCGTATGCAGCCGCCGCATTGCATCAGCCATGATGGATACTTTCTCGGCAGGAGCAAGGTGAGATGACAGGAGATCCTGCCCCGGCACTTCGCCCAATAGCAGCCAGTCCCTTCCCGCTTCAGTGACAACGTCGAGCACAGCTGCGCAAGGAACGCCCGTCGTGGCCAGCCACGATAGCCGCGCTGCCTCGTCCTGCAGTTCATTCAGGGCACCGGACAGGTCGGTCTTGACAAAAAGAACCGGGCGCCCCTGCGCTGACAGCCGGAACACGGCGGCATCAGAGCAGCCGATTGTCTGTTGTGCCCAGTCATAGCCGAATAGCCTCTCCACCCAAGCGGCCGGAGAACCTGCGTGCAATCCATCTTGTTCAATCATGCGAAACGATCCTCATCCTGTCTCTTGATCAGATCTTGATCCCCTGCGCCATCAGATCCTTGGCGGCAAGAAAGCCATCCAGTTTACTTTGCAGGGCTTCCCAACCTTACCAGAGGGCGCCCCAGCTGGCAATTCCGGTTCGCTTGCTGTCCATAAAACCGCCCAGTCTAGCTATCGCCATGTAAGCCCACTGCAAGCTACCTGCTTTCTCTTTGCGCTTGCGTTTTCCCTTGTCCAGATAGCCCAGTAGCTGACATTCATCCGGGGTCAGCACCGTTTCTGCGGACTGGCTTTCTACGTGTTCCGCTTCCTTTAGCAGCCCTTGCGCCCTGAGTGCTTGCGGCAGCGTGAGGGGATCTTGAAGTTCCTATTCCGAAGTTCCTATTCTCTAGAAAGTATAGGAACTTCGAAGCAGCTCCAGCCTACAGGATCCTCTAGAGTCGACCTGCAGGCATGCAAGCTTGGCACTGGCCGTCGTTTTACAACGTCGTGACTGGGAAAACCCTGGCGTTACCCAACTTAATCGCCTTGCAGCACATCCCCCTTTCGCCAGCTGGCGTAATAGCGAAGAGGCCCGCACCGATCGCCCTTCCCAACAGTTGCGCAGCCTGAATGGCGAATGGCGCCTGATGCGGTATTTTCTCCTTACGCATCTGTGCGGTATTTCACACCGCATATGGTGCACTCTCAGTACAATCTGCTCTGATGCCGCATAGTTAAGCCAGCCCCGACACCCGCCAACACCCGCTGACGCGCCCTGACGGGCTTGTCTGCTCCCGGCATCCGCTTACAGACAAGCTGTGACCGTCTCCGGGAGCTGCATGTGTCAGAGGTTTTCACCGTCATCACCGAAACGCGCGA
