# Supplementary figures and images for: The N-Terminal Membrane-Spanning Domain of the Escherichia coli DNA Translocase FtsK Hexamerizes at Midcell
Source: mBio. 2013 Dec 3;4(6):e00800-13. doi: 10.1128/mBio.00800-13 (PMC3870252; doi:10.1128/mBio.00800-13)

A

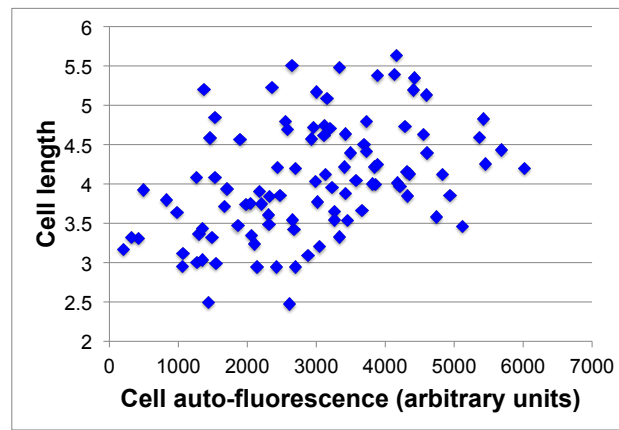

B

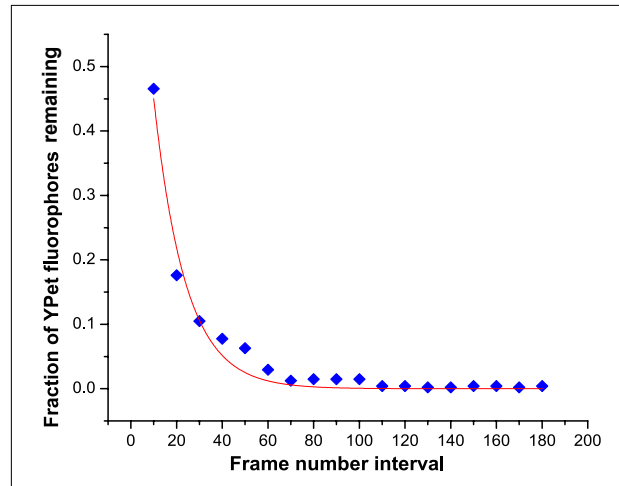

C

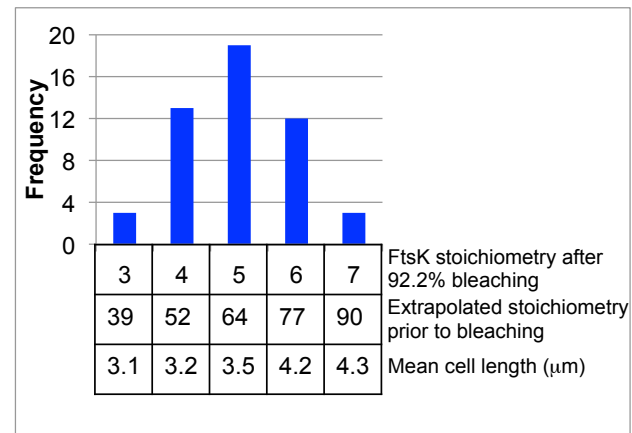

D

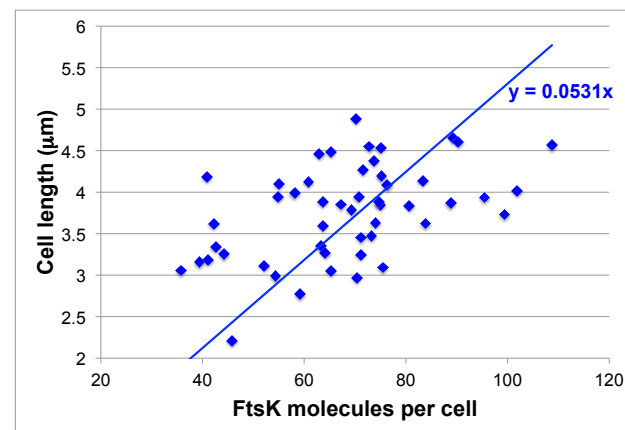

Figure S1 Bisicchia et al.

Supplement: Figure S1 — Calculation of the number of FtsK molecules present at midcell and in whole cells. (A) Auto-fluorescence of wild-type cells as a function of cell length. (B) Plot of the fraction of LacY-YPet fluorophores remaining as a function of time during bleaching. Movies of 477 LacY-YPet molecules were analyzed. The red line shows the single exponential fit. Each frame was 0.0175 s. (C) Number of FtsK molecules in nondividing cells calculated by “bleaching analysis.” A histogram representing the distribution of FtsK copy number observed after bleaching of 92.2% of the initial signal is reported, and the corresponding extrapolated copy number prior to bleaching and mean cell length values are displayed. (D) Numbers of FtsK molecules in nondividing cells as a function of cell length, calculated by “snapshot analysis,” plotted against cell length. The best linearly fitted line is shown. Download [file mbo006131685sf01.pdf]

A

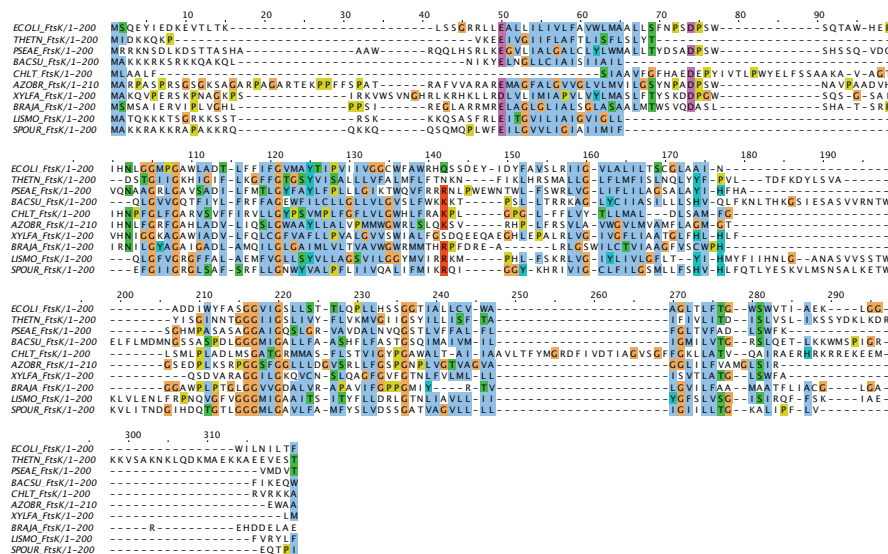

B

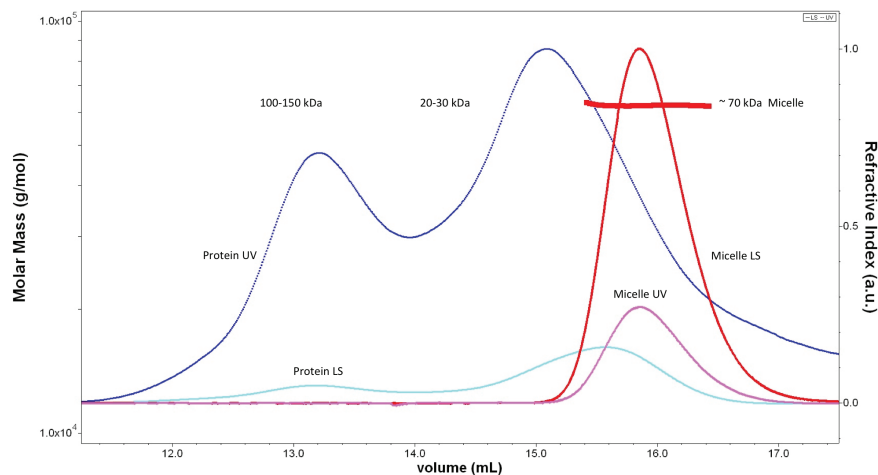

Figure S2 Bisicchia et al.

Supplement: Figure S2 — (A) Multiple-sequence alignment of E. coli, Thermoanaerobacter tengcongensis (TtFtsKN), and 8 other FtsK homologues to show overall sequence conservation in the N-terminal domain, studied here. The alignment was calculated with T-COFFEE using default parameters. FtsK sequences used were Pseudomonas aeruginosa, Bacillus subtilis, Chlorobium tepidum, Azospirillum brasilense, Xylella fastidiosa, Bradyrhizobium japonicum, Listeria monocytogenes, and Sporosarcina ureae. (B) SEC-MALS of TtFtsKN and DDM detergent, showing the same experiment as Fig. 3C. For clarity reasons, the traces resulting from injection of pure detergent were omitted from Fig. 3C and are shown here for completeness. The DDM detergent forms ~70-kDa micelles under the conditions used. Download [file mbo006131685sf02.pdf]

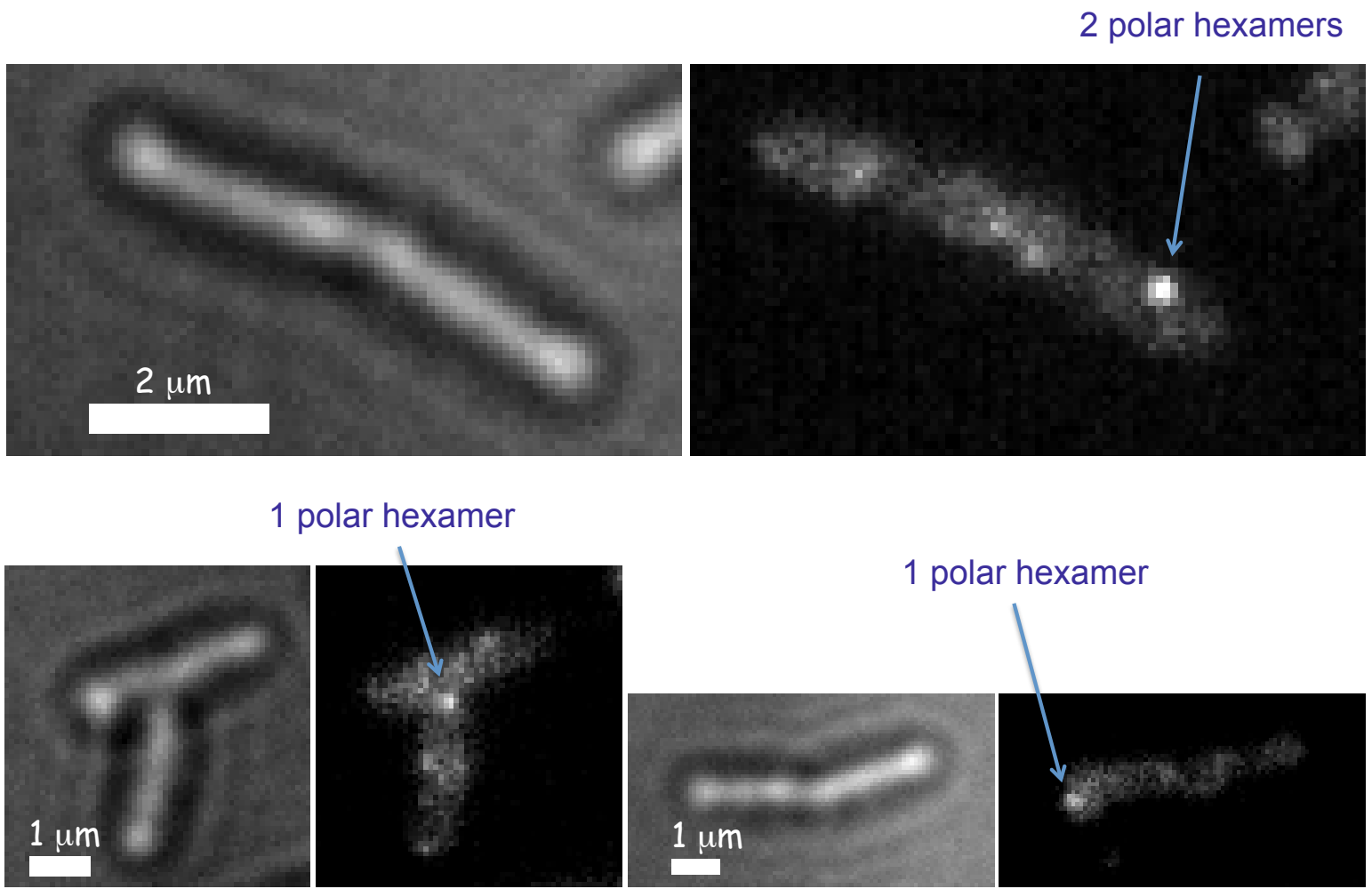

Figure S3 Bisicchia et al.

Supplement: Figure S3 — Noncentral polar FtsK hexamers. DIC and fluorescent images are displayed, and the number of FtsK hexamers is indicated. Download [file mbo006131685sf03.pdf]

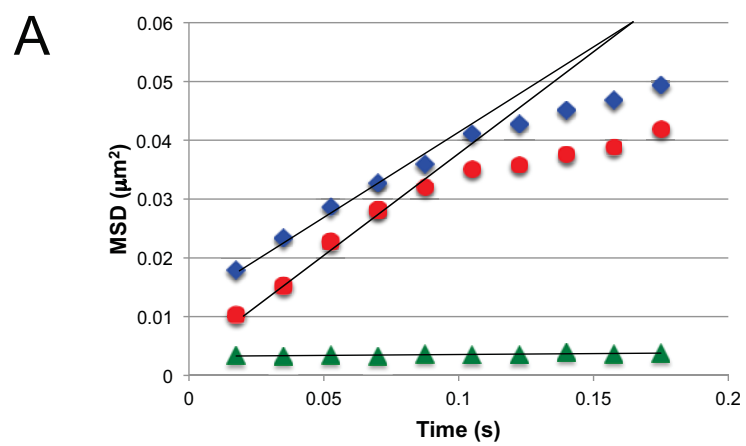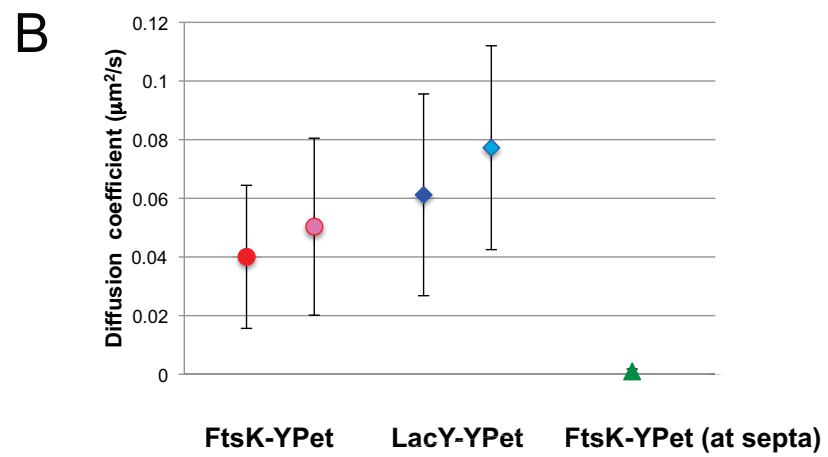

Figure S4 Bisicchia et al.

Supplement: Figure S4 — Diffusion of FtsK-YPet and LacY-YPet. (A) Mean-square displacement [MSD(r2)] as a function of time for noncentral FtsK-YPet single fluorophores (red symbols), LacY-YPet single fluorophores (blue symbols), and FtsK-YPet single fluorophores present in hexamers at midcell (green symbols). The black lines are the linearly fitted over the first four data points in the case of FtsK-YPet and LacY-YPet located outside the cell center and over the whole data set in the case of FtsK-YPet at midcell. (B) Diffusion coefficients of FtsK-YPet molecules outside the cell center (red and pink symbols), LacY-YPet molecules (dark blue and light blue symbols), and FtsK-YPet molecules at midcell (green symbol) as measured by single-particle tracking. The mean value and the standard deviation for 30 independent measurements are displayed. Diffusion coefficient values represented by red, dark blue, and green symbols were obtained by acquiring movies at 10-ms exposure times and 10% laser power, while values represented by pink and light blue symbols were obtained from movies acquired using 1-ms exposure times and 100% laser power. Download [file mbo006131685sf04.pdf]
