# Supplementary material for: The N-Terminal Membrane-Spanning Domain of the Escherichia coli DNA Translocase FtsK Hexamerizes at Midcell
Source: mBio. 2013 Dec 3;4(6):e00800-13. doi: 10.1128/mBio.00800-13 (PMC3870252; doi:10.1128/mBio.00800-13)
Supplement: Table S1 — E. coli K-12 AB1157 strains used in this work. [file mbo006131685st1.docx]

| PB85 | *ftsK-yPet,* Km^R^ | This study |
| --- | --- | --- |
| PB107 | *yPet-ftsQ* | This study |
| PB166 | *zapC-yPet* | This study |
| PB178 | *tolQ-yPet* | This study |
| PB355 | *lacY-yPet,* Km^R^ | This study |
| PB356 | *ftsK_ΔC_-yPet,* Cm^R^ Km^R^ | This study |
| PB391 | *ftsK_N_-yPet,* Cm^R^ Km^R^ | This study |
