# Supplementary material for: The N-Terminal Membrane-Spanning Domain of the Escherichia coli DNA Translocase FtsK Hexamerizes at Midcell
Source: mBio. 2013 Dec 3;4(6):e00800-13. doi: 10.1128/mBio.00800-13 (PMC3870252; doi:10.1128/mBio.00800-13)
Supplement: Table S2 — Oligonucleotides used in this work. [file mbo006131685st2.docx]

| FtsK-F | CAACGGTAATCGTGAAGTGCTGGCCCCACCGCCGTTTGACAGC TCGGCTGGCTCCGCTG |
| --- | --- |
| FtsK-R | TGCGACGCTACCGCGCCTTATCCGGCATACGATGCATTAGCATA TGAATATCCTCCTTAG |
| FtsK-R-cm | CCGTCACAGGTATTTATTCGGCGCAAAGTGCGTTCTAGACATATGA ATATCCTCCTTAG |
| LacY-F | CCGCTTTCCCTGCTGCGTCGTCAGGTGAATGAAGTCGCTTCGGCTGGCTCCGCTGCTGG |
| LacY-R | GTCGGATAAGGCGCTCGCGCCGCATCCGACATTGATTGCCTTATGAATATCCTCCTTAGGCAATCAATGTCGGATGCGGCGCGAGCGCCTTATCCGAC |
| FtsKFX96-F | CTCACCTTCGCCAGTAATCGTACCCGTCGCGATGATACTAGTAGC TCGGCTGGCTCCGCTG |
| FtsKFX96-R | CCGTCACAGGTATTTATTCGGCGCAAAGTGCGTTCTAGAGCGCATATGAATATCCTCCTTAG |
| FtsQ-F | TCGCAGTTGGTAGTACGAATTCTGGAACTGGCGGACTAATTGTAGGCTGGAGCTGCTTCG |
| FtsQ-R | CTCTTCTTCGCTGTTTCGCGTGTTCAGAGCAGCCTGCGACGCGCTGCCAGAACCAGCGGC |
| ZapC-F | GAAACCGCAGGTTAATGTTGACAGCTTCAGCCTCGAACAGGCAGTCAGCTCGGCTGGCTCCGCTG |
| ZapC-R | TCTTTGCTGTAGCTGTGTACCGAAGACTGCACTTAAGTTGGCGCGTTAGCATATGAATATCCTCCTTAG |
| TolQ-F | GCACCGCCAGGCGTTTACCGTTAGCGAGAGCAACAAGGGGAGCTCGGCTGGCTCCGCTG |
| TolQ-R | TGAGATCGCGACGACCTCGTCCACGCGCTCTGGCCATATGTGACCTCCTTACATATGAATATCCTCCTTAG |
